# Increased Glycolysis and Higher Lactate Production in Hyperglycemic Myotubes

**DOI:** 10.3390/cells8091101

**Published:** 2019-09-18

**Authors:** Jenny Lund, D. Margriet Ouwens, Marianne Wettergreen, Siril S. Bakke, G. Hege Thoresen, Vigdis Aas

**Affiliations:** 1 Section for Pharmacology and Pharmaceutical Biosciences, Department of Pharmacy, University of Oslo, 0316 Oslo, Norway; 2 German Diabetes Center (DDZ), Leibniz Center for Diabetes Research, Heinrich Heine University, Medical Faculty, 40225 Düsseldorf, Germany; 3 German Center for Diabetes Research (DZD), 85764 Munich-Neuherberg, Germany; 4 Department of Endocrinology, Ghent University Hospital, B-9000 Ghent, Belgium; 5 Department of Life Sciences and Health, Faculty of Health Sciences, OsloMet - Oslo Metropolitan University, 0130 Oslo, Norway; 6 Center of Molecular Inflammation Research and Department of Clinical and Molecular Medicine, Norwegian University of Science and Technology, 7491 Trondheim, Norway; 7 Department of Pharmacology, Institute of Clinical Medicine, University of Oslo, 0316 Oslo, Norway

**Keywords:** hyperglycemia, myotubes, glycolysis, lactate, substrate oxidation, pathway analysis

## Abstract

Previous studies have shown that chronic hyperglycemia impairs glucose and fatty acid oxidation in cultured human myotubes. To further study the hyperglycemia-induced suppression of oxidation, lactate oxidation, mitochondrial function and glycolytic rate were evaluated. Further, we examined the intracellular content of reactive oxygen species (ROS), production of lactate and conducted pathway-ANOVA analysis on microarray data. In addition, the roles of the pentose phosphate pathway (PPP) and the hexosamine pathway were evaluated. Lactic acid oxidation was suppressed in hyperglycemic versus normoglycaemic myotubes. No changes in mitochondrial function or ROS concentration were observed. Pathway-ANOVA analysis indicated several upregulated pathways in hyperglycemic cells, including glycolysis and PPP. Functional studies showed that glycolysis and lactate production were higher in hyperglycemic than normoglycaemic cells. However, there were no indications of involvement of PPP or the hexosamine pathway. In conclusion, hyperglycemia reduced substrate oxidation while increasing glycolysis and lactate production in cultured human myotubes.

## 1. Introduction

Sustained hyperglycemia in patients with metabolic syndrome contributes both to the diabetic state and to diabetic complications [1,2,3,4]. This glucose toxicity is thought to be mediated by an increased flux of glucose through the glycolytic pathway, generation of glycosylated proteins, and reactive oxygen species (ROS) [5].

We have previously shown that chronic hyperglycemia (HG), compared to normoglycemia (NG), affected cultured skeletal muscle cells from healthy volunteers by reducing energy substrate utilization, increasing triacylglycerol storage, and impairing metabolic switching [6,7]. We also observed that the addition of lactate to the cells induced similar effects as hyperglycemia on glucose and oleic acid (OA) metabolism [6]. This effect of lactate was specific, as other acids that lowered pH to the same extent did not affect glucose and OA metabolism. Increased production of lactate could be a factor in the metabolic changes observed in HG-treated cells. The increase in lactate after HG may be a consequence of impaired mitochondrial function, or, simply, the result of excess glucose increasing glycolytic flux to an extent that preferentially shunts pyruvate to lactate instead of entering the mitochondria.

Under hyperglycemic conditions, more glucose will flow through the glycolytic pathway and pathways that branch off from it [8], thereby increasing flux into pathways that normally are of minor importance such as the hexosamine and the pentose phosphate pathway (PPP). The hexosamine pathway generates glucosamine 6-phosphate that is further converted to uridine diphosphate *n*-acetylglucosamine, which is a substrate for *o*-GlcNac transferases that catalyzes glycosylation reactions of proteins. The hexosamine pathway has also been suggested to be involved in ROS production and diabetic complications [9]. PPP is an important provider of precursors to amino acid and nucleotide biosynthesis, as reductants (i.e., NADPH) for biosynthetic anabolic processes, including fatty acid synthesis. Another role of the PPP is to maintain carbon homeostasis and the redox potential necessary to protect against oxidative stress. High glycolytic flux will also generate high levels of NADH in cells, and, thereby, increase the pressure on the electron transport chain, particularly complex I [8]. Consequently, the production of superoxide and other ROSs will increase and might mediate glycotoxic effects.

Chronically elevated blood lactate levels have been found in patients with type 2 diabetes [10]. Lactate itself can downregulate enzyme activity in the glycolytic process, as hexokinase and phosphofructokinase [11], and long-term lactate infusion in rats has been shown to reduce glucose uptake in skeletal muscles by downregulating the protein expression of glucose transporter (GLUT) 4 [12].

The purpose of the present study was to further examine the hyperglycemia-induced suppression of substrate oxidation in cultured human myotubes.

## 2. Materials and Methods

### 2.1. Materials

DMEM-Glutamax™ low glucose, heat-inactivated FBS, DPBS with and without Mg^2+^ and Ca^2+^, penicillin/streptomycin (10000 IE/mL), amphotericin B, and trypsin-EDTA were from Gibco Invitrogen (Gibco, Life Technologies, Paisley, UK). Ultroser G was from Pall (Cergy-Saint-Christophe, France) and insulin (Actrapid^®^ Penfill^®^ 100 IE/mL) was from Novo Nordisk (Bagsvaerd, Denmark). SkBM-kit (SkGM) was from Lonza (Walkersville, MD, USA). BSA (essentially fatty acid-free), d-glucose, sodium lactic acid, HEPES, DMSO, l-glutamine, azaserine, PUGNAc, 6-aminonicotinamide (6-AN), MitoTEMPO, and cytochalasin B were from Sigma-Aldrich (St. Louis, MO, USA). d-[^14^C-(U)]glucose (107 MBq or 185 MBq) and l-[^14^C(U)]-lactic acid (1.85 MBq) were from PerkinElmer (Boston, MA, USA). Corning^®^ CellBIND^®^ tissue culture plates and flasks were from Corning Life Sciences (Schiphol-Rijk, the Netherlands). Unifilter^®^-96 GF/B, Isoplate^®^-96, Optiphase Supermix, and TopSeal-A transparent film were from Perkin Elmer (Shelton, CT, USA). Bio-Rad Protein Assay Dye Reagent Concentrate, Clarity™ Western ECL Substrate, 10% Mini-PROTEAN^®^ TGX™ gels, Precision Plus Protein Standard, and Immun-Blot^®^ polyvinylidene difluoride (PVDF) Membranes were from Bio-Rad (Copenhagen, Denmark). OxiSelect intracellular ROS assay kit was from Cell Biolabs (San Diego, CA, USA). The *O*-GlcNAc Monoclonal Antibody (#CTD110.6) was from BioLegend (San Diego, CA, USA). Goat Anti-Mouse IgM-HRP conjugated antibody was from Southern Biotech (Birmingham, AL, USA). A549 Cell Lysate positive control (#sc-2413) was from Santa Cruz Biotechnology (Santa Cruz, CA, USA). Seahorse XF Base medium and Seahorse XF Cell Mito Stress Test Kit were from Agilent Technologies (Santa Clara, CA, USA).

### 2.2. Ethics Statement

The muscle biopsies were obtained with informed written consent and approval by the National Committee for Research Ethics, Oslo, Norway (approvals S-04133 and 2011/2207). The study adhered to the Declaration of Helsinki.

### 2.3. Cell Culturing

Multinucleated human myotubes were established by activation and proliferation of satellite cells isolated from musculus obliquus internus abdominis from 11 healthy donors (six male, five female), age 44.0 ± 3.9 years, BMI 23.3 ± 1.0 kg/m^2^, fasting glucose 5.2 ± 0.2 mM, insulin, plasma lipids, and blood pressure within normal range and no family history of diabetes. For the MitoTEMPO experiments satellite cells were isolated from musculus vastus lateralis from four healthy male volunteers, age 24 ± 1.1 years, BMI 21.4 ± 1.1 kg/m^2^, fasting glucose 4.7 ± 0.1 mM, insulin, plasma lipids, and blood pressure within normal range and no family history of diabetes (complete set of donor characteristics has been reported previously [13]). Clonetics human myoblasts (Lonza, Cologne, Germany) isolated from three healthy donors (one male, two female), age 32.3 ± 6.3 years, were used to measure the oxygen consumption rate and proton exchange rate. 

For the proliferation of myoblasts, a DMEM-Glutamax medium (5.5 mM glucose) supplemented with 2% FBS, 2% Ultroser G, penicillin/streptomycin, and amphotericin B were used. At 70–80% confluence the medium was replaced by a DMEM-Glutamax medium with 5.5 mM glucose (NG) supplemented with 2% FBS, penicillin/streptomycin, amphotericin B, and 25 pM insulin to induce differentiation. The last 4 days of the differentiation period cells for hyperglycemia were treated with 20 mM glucose (HG) with or without l-glutamine (0–20 mM), PUGNAc (100 µM), azaserine (20 µM), 6-AN (50 µM), or MitoTEMPO (10 nM). The cells were cultured in a humidified 5% CO_2_ atmosphere at 37 °C, and the medium was changed every 2–3 days. Experiments were performed after 7 days of differentiation.

As the medium was always filtrated for sterilization we compared unfiltered and filtered media to ensure that this did not affect the concentration of substances within the media, which again resulted in possible differences between HG and NG myotubes. No differences were observed using filtered versus unfiltered media (data are available upon request). Furthermore, as it may be hypothesized that chronic hyperglycemia may induce hypertonicity in the medium, we explored this possibility by comparing NG with normal HG induced by d-glucose, HG induced by l-glucose, and similar concentration of mannitol (15 mM) on glucose uptake and oxidation in the myotubes. The observed effects are due to the HG treatment and not hypertonicity (data are available upon request). 

### 2.4. Substrate Oxidation Assay

Skeletal muscle cells (7000 cells/well) were cultured on 96-well CellBIND^®^ microplates. Substrates, d-[^14^C(U)]glucose (0.58 or 1 µCi/mL, 111 or 200 μM) or l-[^14^C(U)]-lactic acid (50 µCi/mL, 100 µM) were given with or without 5 mM glucose during 4 h CO_2_ trapping as previously described [14]. In brief, a 96-well UniFilter^®^-96 GF/B microplate was mounted on top of the CellBIND^®^ plate, and CO_2_ production was measured in the DPBS medium with 20 mM HEPES adjusted to pH 7.2–7.3. CO_2_ production and cell-associated (CA) radioactivity were assessed using a PerkinElmer 2450 MicroBeta^2^ scintillation counter. Protein levels in the lysate were measured with the Bio-Rad protein assay using a VICTOR™ *X*4 Multilabel Plate Reader. The sum of ^14^CO_2_ and remaining CA radioactivity was taken as a measurement of total cellular uptake of the substrate.

### 2.5. Mitochondrial Stress Assay

Oxygen consumption rates (OCR) and extracellular acidification rates (ECAR) were recorded in primary human myotubes using a Seahorse XF24e analyzer. One hour before the start of the recordings, the medium was changed to Seahorse XF Base medium, supplemented with 5 mM glucose, 2 mM glutamine, 1 mM sodium pyruvate, 0.5 mM HEPES, and 80 µg/mL carnitine, pH 7.4. Then, OCR was recorded three times at 6 min intervals at baseline, and followed by injections with 5 µM oligomycin (Oli), 3 µM carbonyl cyanide 4-(trifluoromethoxy)phenylhydrazone (FCCP), and 4 µM rotenone/antimycin A (Rot/AA) (XF Cell Mito Stress Test Kit, Agilent Technologies), respectively. Determinants of mitochondrial function (basal respiration, proton leak, maximal respiration, spare respiratory capacity, non-mitochondrial oxygen consumption, and ATP production) were calculated using the following formulas: Basal respiration = last rate measurement before the first injection–non-mitochondrial respiration rate; proton leak = minimum rate measurement after Oli injection–non-mitochondrial respiration; maximal respiration = maximum rate measurement after FCCP injection–non-mitochondrial respiration; spare respiratory capacity = maximal respiration–basal respiration; non-mitochondrial oxygen consumption = minimum rate measurement after Rot/AA injection; ATP production = last rate measurement before Oli injection–minimum rate measurement after Oli injection. ATP production was converted to mitochondrial ATP production rate by the formula: mitoATP production rate = ATP production × 2 (pmol O/pmol O_2_) × P/O (pmol ATP/pmol O), whereby P/O was set at a validated value of 2.75 [15]. The conversion of ECAR into proton efflux rate (PER) [15,16,17] was performed to calculate the glycolytic ATP production rate. The glycolytic ATP production rate is equivalent to the glycolytic proton efflux rate (glycoPER), which, in turn, is obtained by subtracting mitochondrial PER from total PER as described [17].

### 2.6. Glycolytic Rate Analysis

For glycolytic rate analysis in primary human myotubes, the medium was changed to Seahorse XF Base medium, supplemented with 5 mM glucose, 2 mM glutamine, 1 mM sodium pyruvate, and 0.5 mM HEPES, pH 7.4. Then, ECAR and OCR were recorded using the Seahorse XF24e analyzer, six times at 6 min intervals at baseline, and following injections with 4 µM Rot/AA and 50 mM 2-deoxyglucose (2-DG) (XF Cell Mito Stress Test Kit), respectively. PER, glycoPER, basal glycolysis, basal proton efflux rate, compensatory glycolysis, and post 2-DOG acidification were calculated as previously described [17,18].

### 2.7. Measurement of ROS Concentration

The intracellular content of ROS was measured with the OxiSelect Intracellular ROS assay kit (Cell Biolabs) according to the manufacturer’s instructions. Hydrogen peroxide (H_2_O_2_) was used as a positive control.

### 2.8. Measurement of Lactate Concentration

Cell media were collected at the end of the differentiation period before substrate oxidation measurements. Lactate was measured in 15 µL of the cell media with Accutrend Plus (Roche Diagnostics).

### 2.9. Immunoblotting

Myotubes were cultured in 6-well plates. The last 4 days of the differentiation period cells were under NG or HG conditions with or without azaserine (20 µM) or PUGNAc (100 µM). The samples for immunoblotting were harvested in a sample buffer and the protein was measured. Aliquots of 15 µg cell protein from total cell lysates, including a positive control (cell lysate A549) were electrophoretically separated on 10% polyacrylamide gels, followed by blotting to PVDF membranes. The membranes were incubated with an antibody against anti-*o*-GlcNAc (1:1000) overnight. Immunoreactive bands were visualized with enhanced chemiluminescence. Three separate experiments (n = 3) were performed.

### 2.10. Microarray Analysis

A method for RNA isolation and microarray analysis has been described previously [6]. In short, human myotubes were treated with or without HG as described in Section 2.3, the RNA was isolated and run on an Illumina HumanWG-6 v3.0 expression BeadChip. Gene expression was analyzed using the data available at the gene expression omnibus (GEO) at http://www.ncbi.nlm.nih.gov/geo/ with accession number GSE19620.

Pathway-ANOVA analysis was performed in Partek Genomics 6.6 and Partek Pathway using pathways from the Kyoto Encyclopedia of Genes and Genomes (KEGG) database and with the treatment and donor factors included in the analysis. Pathway-ANOVA found 79 differentially expressed pathways (FDR *p* < 0.05) between HG and NG in the data set imported from GEO.

### 2.11. Statistics

Data are presented as means ± SEM. The value *n* represents the number of different donors, each with at least duplicate observations. Statistical comparison between different treatments was performed by paired Student’s t-test using GraphPad Prism 8 for Windows. The Seahorse data were analyzed by an unpaired Student’s t-test using GraphPad Prism 8 for Mac. Differences were considered statistically significant at *p*-values < 0.05. To reduce the chance of false positives, an FDR-adjusted *p*-value (or *q*-value) was used for the pathway-ANOVA analysis; FDR *p* < 0.05 was considered statistically significant.

## 3. Results

### 3.1. Effect of Chronic Hyperglycemia on Glucose and Lactic Acid Oxidation

**Figure 1:**
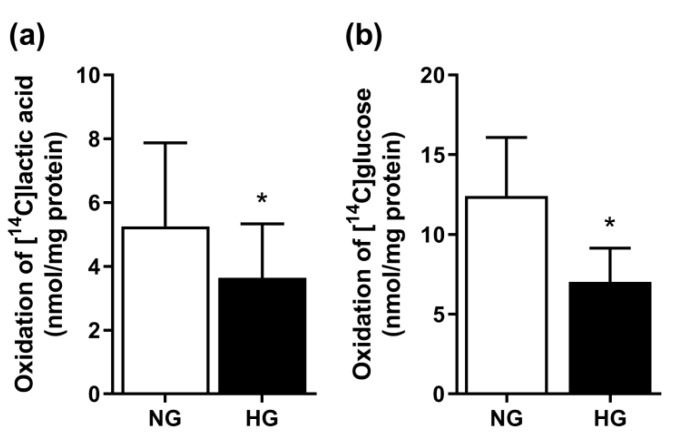
Effects of hyperglycemia on lactic acid and glucose oxidation. Myotubes were exposed to 20 mM glucose (HG) or standard differentiation medium (NG, 5.5 mM glucose) the last 4 days of the differentiation period, and then incubated with either [^14^C(U)]lactic acid (1 µCi/mL, 100 µM) or d-[^14^C(U)]glucose (0.5 µCi/mL, 200 µM) for 4 h. Oxidation was measured as CO_2_ trapped in a filter and counted by liquid scintillation. (**a**) Lactic acid oxidation after chronic HG. Results are presented as means ± SEM in nmol/mg protein from five individual experiments (n = 5). (**b**) Glucose oxidation after chronic HG. Results are presented as means ± SEM in nmol/mg protein from 15 individual experiments (n = 15). * Statistically significant vs. NG (*p* < 0.05, paired Student’s *t*-test).

In previous studies, four days treatment of the cells with 20 mM glucose was used as chronic hyperglycemic (HG) conditions [6,7], and we observed that HG compared to normoglycemia (NG, 5.5 mM glucose) impaired both glucose and fatty acid oxidation, where glucose metabolism was the most affected [6]. We hypothesized that substrate oxidation, in general, was suppressed by HG and, therefore, studied the oxidation of lactic acid, another energy source in skeletal muscle cells [19]. Mean lactic acid oxidation was reduced by 31% in myotubes treated with HG compared to NG (Figure 1a). The previously observed effect of HG on glucose oxidation [6] was confirmed; chronic HG reduced glucose oxidation by 43% compared to NG (Figure 1b). 

### 3.2. Effect of Hyperglycemia on Mitochondrial Function

**Figure 2:**
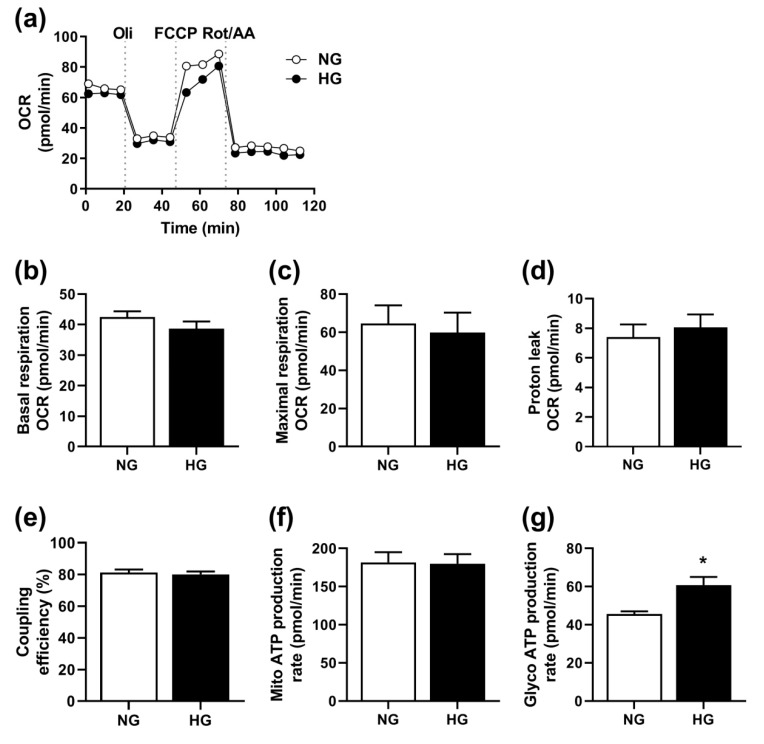
Effects of chronic hyperglycemia on mitochondrial function. Human skeletal muscle cells were grown in 24-well Seahorse tissue culture plates, exposed to 20 mM glucose (HG) for the last four days of the differentiation period, or standard differentiation medium (NG, 5.5 mM glucose), before measurement of the oxygen consumption rate (OCR) with the Seahorse XF24e analyzer. OCR was recorded three times at 6 min intervals at baseline, and following injections with 5 µM oligomycin (Oli), 3 µM FCCP and 4 µM rotenone/antimycin A (Rot/AA), respectively (XF Cell Mito Stress Test Kit). Determinants of mitochondrial function were calculated as described in Section 2.5. (**a**) One representative experiment. (**b**–**g**) Mean ± SEM from five individual experiments (n = 5). (**b**): basal respiration calculated from OCR in pmol/min, (**c**): maximal respiration calculated from OCR in pmol/min, (**d**): proton leak calculated from OCR in pmol/min, (**e**): percentage coupling efficiency, (**f**): mitochondrial (Mito) ATP production rate in pmol/min, and (**g**): glycolytic (Glyco) ATP production rate in pmol/min. * Statistically significant vs. NG (*p* < 0.05, unpaired Student’s *t*-test).

Based on the effects of chronic hyperglycemia on substrate oxidation, we hypothesized that hyperglycemia impaired mitochondrial function. Therefore, we measured mitochondrial function by assessing OCR (Figure 2a) using a Seahorse XF Analyzer. No significant differences between NG- and HG-treated myotubes were observed, neither in basal respiration (Figure 2b), maximal respiration (Figure 2c), proton leak (Figure 2d), coupling efficiency (Figure 2e), nor mitochondrial ATP production (Figure 2f), indicating no effect of HG on mitochondrial function. Yet, glycolytic ATP production calculated from the Mito Stress Test was increased by HG (Figure 2g).

### 3.3. Involvement of ROS in Hyperglycemia

**Figure 3:**
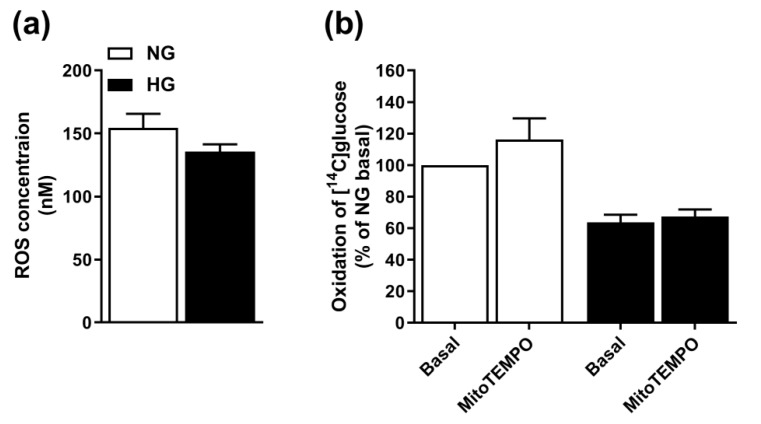
Involvement of reactive oxygen species (ROS). (**a**) Myotubes were exposed to 20 mM glucose (hyperglycemia, HG) the last four days of the differentiation period and ROS production in HG versus normoglycaemic (NG) myotubes were calculated from a standard curve (OxiSelect intracellular ROS assay kit). Results are presented as means ± SEM from five individual experiments (n = 5). (**b**) Effect of the ROS-scavenger MitoTEMPO on glucose oxidation. Results are presented as means ± SEM in % of NG basal from four individual experiments (n = 4).

We also considered the role of ROS for the observed hyperglycemia-suppression of oxidation. The ROS level in HG-treated cells was 90.5% (±9.4) of the level in NG-treated cells, indicating that the effect of HG was not mediated by increased ROS production (Figure 3a). Furthermore, the effect of HG on glucose oxidation was not reversed by the ROS-scavenger MitoTEMPO (Figure 3b), thus, indicating that ROS is not responsible for the HG-induced effect.

### 3.4. Effects of Hyperglycemia on Gene Expression

**Figure 4:**
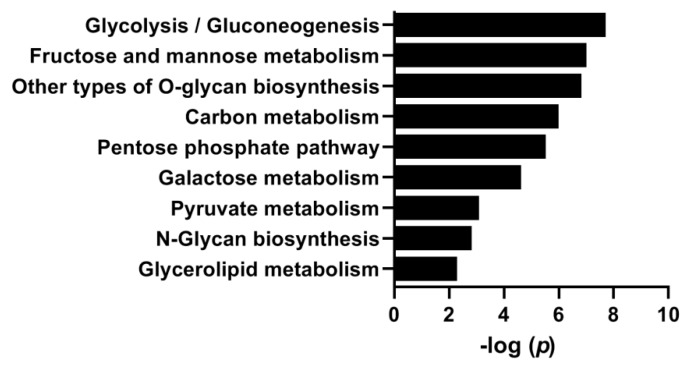
Pathways involved in metabolic processes regulated by hyperglycemia. Myotubes were exposed to 20 mM glucose (HG) or normal glucose concentration (NG) for the last four days of the differentiation period. Gene expression was measured using the Illumina HumanWG-6 v3.0 expression BeadChip (microarray). Pathway-ANOVA was performed to identify pathways regulated by HG compared to NG in myotubes from three donors (n = 3). Selected significantly (FDR *p* < 0.05) regulated pathways with relation to carbohydrate metabolism are presented.

To examine the effect of HG on gene expression, we reanalyzed a previously performed microarray [6]. When analyzing the data by pathway-ANOVA and focusing on pathways related to carbohydrate metabolism, HG significantly upregulated several metabolic processes and pathways, including glycolysis/gluconeogenesis, PPP, pyruvate metabolism, and *N*-glycan biosynthesis (Figure 4).

### 3.5. Effect of Hyperglycemia on Glycolysis

**Figure 5:**
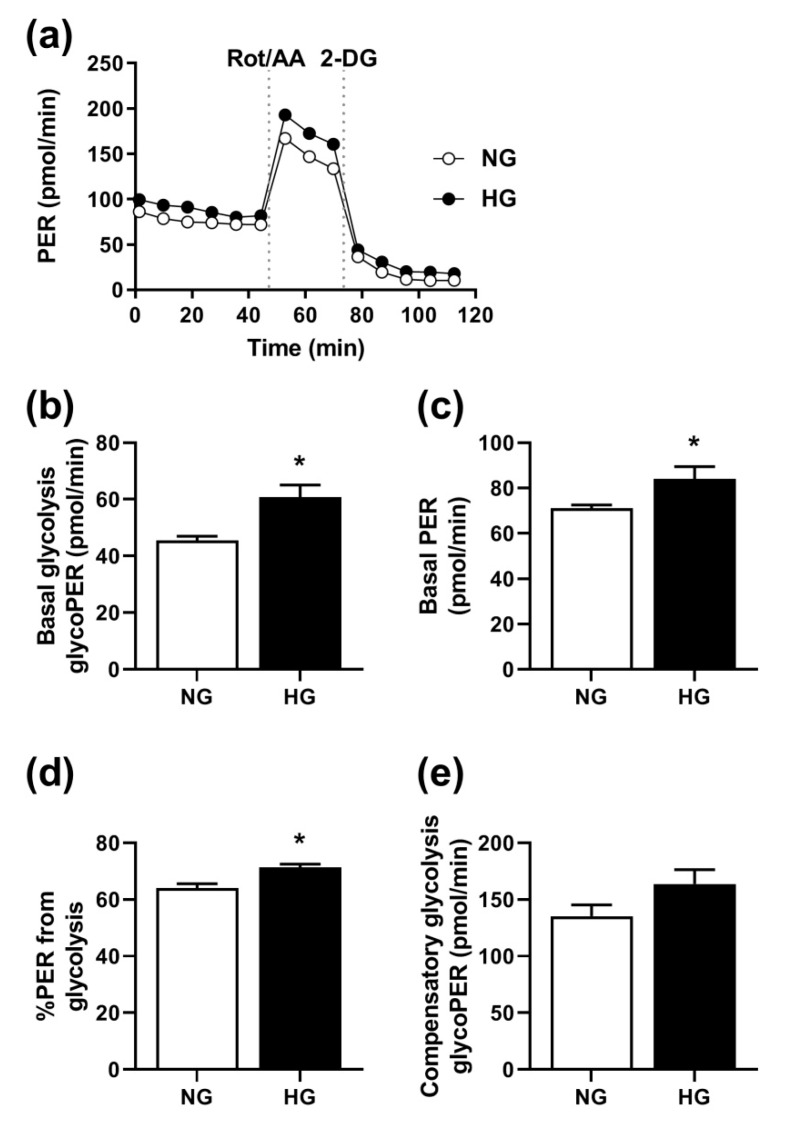
Effects of chronic hyperglycemia on the glycolytic rate. Human skeletal muscle cells were grown in 24-well Seahorse tissue culture plates, exposed to 20 mM glucose (HG) for the last four days of the differentiation period, or the standard differentiation medium (NG, 5.5 mM glucose), before measurement of the glycolytic rate with the Seahorse XF24e analyzer. Extracellular acidification rates and oxygen consumption rates were recorded six times at 6 min intervals at baseline, and following injections with 4 µM rotenone/antimycin A (Rot/AA) and 50 mM 2-deoxyglucose (2-DG), respectively. Proton efflux rate (PER), glycolytic proton efflux rate (glycoPER), basal glycolysis, basal PER, compensatory glycolysis, and post 2-DOG acidification were calculated as described in Section 2.6. (**a**) One representative experiment. (**b**–**e**) Mean ± SEM from five individual experiments (n = 5). (**b**): basal glycolysis glycoPER in pmol/min, (**c**): basal PER in pmol/min, (**d**): percentage PER from glycolysis, and (**e**): compensatory glycolysis glycoPER in pmol/min. * Statistically significant vs. NG (*p* < 0.05, unpaired Student’s *t*-test).

Based on the results from the pathway analysis, we wanted to study some aspects further using functional studies. We used the Seahorse XF24e Analyzer to perform a glycolytic rate assay on living myotubes. As shown in Figure 5, HG increased basal glycolysis, PER, and the PER derived from glycolysis. There was no statistically significant effect of HG on compensatory glycolysis (*p* = 0.07). 

### 3.6. Lactate Concentration in Media

**Figure 6:**
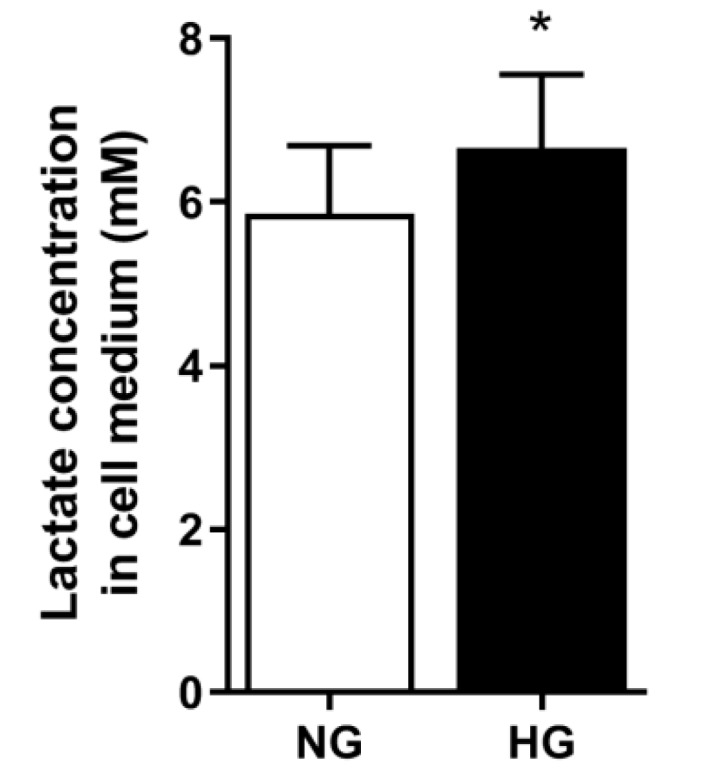
Concentration of lactate in cell media. Myotubes were treated with 20 mM glucose (HG) or 5 mM glucose (NG) for four days. Thereafter, the media was removed and lactate concentration measured using Accutrend Plus. Results are presented as means ± SEM in mM lactate from five individual experiments (n = 5). * Statistically significant vs. NG (*p* < 0.05, paired Student’s *t*-test).

Previously, we have observed that four days pretreatment with 10 mM lactic acid induced the same magnitude of suppression of oxidation as HG [6]. Furthermore, given the observed effect of HG on glycolysis, we also wanted to see if HG affected the lactate concentration. Lactate concentration, measured in the cell media at the end of the four days pretreatment period, was higher in media from HG than from NG myotubes (Figure 6).

### 3.7. Involvement of the PPP in Hyperglycemia

**Figure 7:**
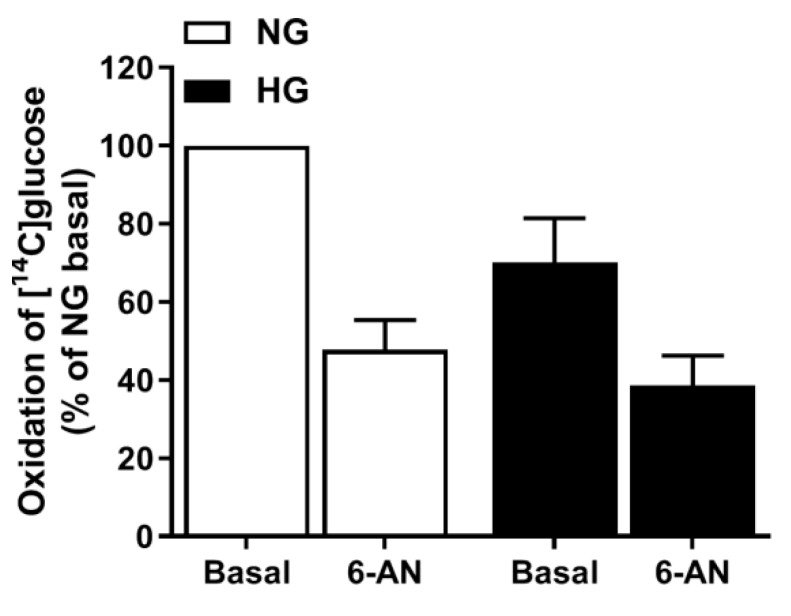
Involvement of the pentose phosphate pathway. Myotubes were exposed to 5.5 mM (NG) or 20 mM glucose (HG) with or without 50 µM 6-aminonicotinamide (6-AN) the last four days of the differentiation period and then incubated with d-[^14^C(U)]glucose (0.5 µCi/mL, 200 µM) for 4 h. Oxidation was measured as CO_2_ trapped in a filter and counted by liquid scintillation. Data are presented as means ± SEM relative to NG basal from four individual experiments (n = 4). Data representing NG basal (100%): 26.6 ± 10.3 nmol/mg protein.

The ANOVA-pathway analysis showed that PPP was upregulated by hyperglycemia (Figure 4); PPP is a metabolic pathway parallel to glycolysis. To functionally evaluate the role of the PPP we inhibited glucose-6-phosphate dehydrogenase, the enzyme involved in the first step of the pathway generating one NADPH, using 6-AN (Figure 7) to see if the suppression by hyperglycemia on glucose oxidation was abolished. However, glucose oxidation was suppressed even further by 6-AN, in both NG and HG myotubes, and thereby did not confirm the result from the pathway analysis. 

### 3.8. Involvement of the Hexosamine Pathway

**Figure 8:**
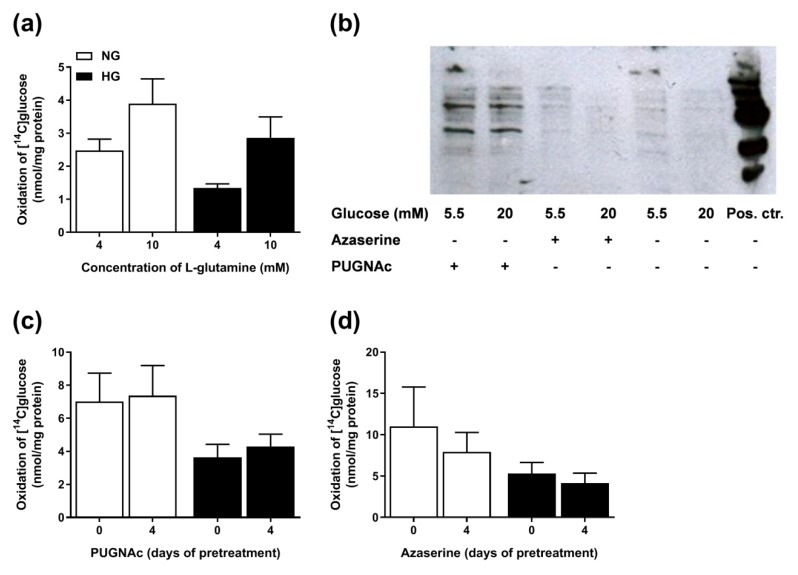
Involvement of the hexosamine pathway. (**a**) Myotubes were treated with 20 mM glucose (HG) or 5 mM glucose (NG) for four days in the presence of 4 mM (basal) or 10 mM l-glutamine before they were incubated with d-[^14^C(U)]glucose (0.5 µCi/mL, 200 µM) for 4 h. Glucose oxidation was measured as CO_2_ trapped in a filter and counted by liquid scintillation. (**b**) Effects of the deglycosylation inhibitor PUGNAc (100 µM) and the glycosylation inhibitor azaserine (20 µM) in protein lysates from HG and NG cells. The inhibitors were added together with HG the last four days of the differentiation period. Aliquots of 15 µg cell protein from total cell lysates, including a positive control (cell lysate A549), were electrophoretically separated on 10% polyacrylamide gels, followed by immunoblotting with specific antibody for *O*-GlcNAc. One representative Western blot from three individual experiments (n = 3) is shown. (**c**) Effect of the deglycosylation inhibitor PUGNAc (100 µM) on glucose oxidation. (**d**) Effect of the glycosylation inhibitor azaserine (20 µM) on glucose oxidation. Results are presented as means ± SEM in nmol/mg protein from three individual experiments (n = 3).

As it has been described that more glucose will flow through alternative metabolic pathways during hyperglycemic conditions, we also wanted to examine the involvement of the hexosamine pathway. The rate-limiting enzyme of this pathway, glucosamine-fructose-6-phosphate amidotransferase (GFAT), uses l-glutamine as a substrate. We hypothesized that if hyperglycemia pretreatment upregulates this pathway then the effect of hyperglycemia could be limited by the concentration of l-glutamine, and possibly the effect of hyperglycemia would be boosted by adding l-glutamine to the cell medium. Therefore, l-glutamine was added to the medium before glucose oxidation was measured (Figure 8a). Glucose oxidation was as expected overall lower in HG myotubes, but no significant effect of l-glutamine was observed, and the tendency to increase glucose oxidation with increasing concentrations of l-glutamine was similar in NG and HG cells (data not shown). The hexosamine pathway generates substrates for *O*-GlcNAc transferases that catalyze the *o*-glycosylation of proteins. To further study this pathway, we looked at the effects of the deglycosylation inhibitor PUGNAc (through inhibition of GlcNAcase) and glycosylation inhibitor azaserine (through inhibition of GFAT) on protein glycosylation using the immunoblotting technology (Figure 8b). No clear differences between NG and HG myotubes were observed, but cells treated with PUGNAc showed increased protein glycosylation compared to untreated cells. Therefore, we also wanted to examine the effects of the enzyme inhibitors on glucose oxidation (Figure 8c,d). No differences were observed in glucose oxidation between NG and HG cells after pretreatment with PUGNAc (Figure 8c) or azaserine (Figure 8d). Combined, the results indicate that the hexosamine pathway most likely is not crucial for the effects of chronic hyperglycemia on energy metabolism.

## 4. Discussion

In this study, we aimed to elucidate the mechanism behind hyperglycemia-induced suppression of glucose oxidation. We observed that HG myotubes not only had lower glucose oxidation, but lactic acid oxidation was also lower compared to NG myotubes. However, further mitochondrial studies by Seahorse did not show any differences between HG and NG myotubes, and ROS were not involved either. Pathway analysis of microarray data indicated that HG regulated several pathways in cultured skeletal muscle cells, including glycolysis/gluconeogenesis, the PPP, galactose metabolism, pyruvate metabolism, *n*-glycan biosynthesis, and glycerolipid metabolism on a gene level. Individual gene expression changes were all minor in response to HG, and, thus, these changes may not have been detrimental for the functional assays performed. The kinetics of enzymes involved in the pathways might also be a factor when performing these assays. Functional studies did not indicate that the hexosamine pathway or PPP were affected by HG. However, glycolysis was found to be higher in HG compared to NG myotubes, as was lactate concentrations in media from HG versus NG cells. We hypothesize that this may, at least in part, explain the HG-induced effects. 

Hyperglycemia significantly contributes to insulin resistance in skeletal muscle [20,21,22,23]. The mechanism behind this glucose toxicity has proven to be quite complex. We have previously observed that HG in cultured human myotubes reduced both basal and insulin-stimulated glucose uptake and glycogen synthesis compared to NG [7]. The incorporation of glucose into cellular lipids was, on the other hand, increased by HG [7]. Furthermore, we have previously observed that HG reduced oxidation of both glucose and OA [6]. We have recently confirmed that skeletal muscle cells can use lactate as an energy source [19], and to explore whether HG induces a general reduction in substrate oxidation by HG, we studied lactic acid oxidation. The present study showed that HG reduced lactic acid oxidation in the myotubes. As oxidation of all three major energy substrates for skeletal muscles were suppressed by hyperglycemia it further indicated that the mitochondrial function might be impaired. 

It is thought that in diabetes the hyperglycemia leads to the overproduction of ROS, as well as changes in mitochondrial morphology and biogenesis [24,25,26]. Palmeira and co-workers showed that the hyperglycemia-induced overproduction of ROS led to a decrease in mitochondrial copy number in HepG2 liver cells, and this decrease in mitochondrial biogenesis was followed by a decrease in transcripts of mitochondrial transcription factor A and, therefore, loss of respiratory efficacy [25]. In aortic valvular interstitial cells, however, chronic HG increased mitochondrial function twofold, indicating cell- or tissue-specific effects [16]. Previously, we speculated that HG leads to a reduced mitochondrial function due to the reduced effect of dinitrophenol, lower concentration of ATP, and a tendency towards a slightly decreased level of mtDNA [6]. No effects of HG on oxidative pathways were found on the gene level; neither could we detect any differences in mitochondrial function between NG- and HG-treated myotubes. In the present work, we have also observed reduced substrate (glucose and lactic acid) oxidation, but direct measurements on cellular respiration with Seahorse revealed no signs of mitochondrial dysfunction per se. Furthermore, the concentration of ROS was similar in NG and HG cells, and the ROS-scavenger MitoTEMPO did not affect glucose oxidation differently in NG and HG myotubes. In cardiomyocytes, the production of ROS increases within minutes after addition of glucose [27]. In our experiments the high glucose load was continuous for four days; therefore, ROS production would also be expected to stay high for the whole period, and still be seen after four days if present. However, it is possible that ROS production could have increased immediately after addition of HG and may not be present in detectable amounts after four days. However, if ROS was crucial for the HG-induced effects it would be expected that MitoTEMPO prevented it (which was added together with HG). Wautier and co-workers, who used cultured vascular endothelial cells as a model for diabetic vascular damage, observed that hyperglycemia led to higher production of superoxide (ROS) [28]. Furthermore, others have shown that hyperglycemia mediates an increase in ROS production due to the increased input of reducing agents to the electron transport chain [9,29]. This was further associated with increased flux of the hexosamine pathway [5,9,30]. However, the present study implies that mitochondrial dysfunction and ROS-production are not crucial for HG-mediated effects in cultured human myotubes.

Chronic hyperglycemia has been associated with aberrant signaling processes mediated by enzymes in the hexosamine pathway [5,9,30,31]. The hyperglycemia-mediated increased production of mitochondrial superoxide in vascular endothelial cells reported by Wautier and co-workers was functionally linked to the shunting of glucose into the hexosamine pathway, resulting in activation of pro-inflammatory signaling processes [28]. Hu and co-workers examined the putative role of altered hexosamine pathway signaling and selective *O*-GlcNAcylation of mitochondrial proteins by inducing hyperglycemia in cultured cardiac myocytes. The hyperglycemia resulted in enhanced *O*-GlcNAcylation of proteins in the electron transport chain, complexes I, III, and IV, and the authors linked this to impaired mitochondrial function in the cells [32]. In the present study, we examined the role of the hexosamine pathway by treatment with l-glutamine, as GFAT, the rate-limiting enzyme of this pathway, uses l-glutamine as a substrate and as it has been proposed as a determining factor in the hyperglycemia-mediated activation of the hexosamine pathway [33]. Furthermore, we looked at the effects of the deglycosylation inhibitor PUGNAc and glycosylation inhibitor azaserine. However, no differences between NG and HG myotubes were observed, and we conclude that the hexosamine pathway most likely is not involved in the hyperglycemia-induced suppression of substrate oxidation.

As the PPP is important for the maintenance of carbon homeostasis and the redox potential necessary to protect against oxidative stress [34,35,36], we also examined its role in the effects of hyperglycemia. Pathway analysis performed on the microarray indicated the role of the PPP in the effects of hyperglycemia; however, we were not able to confirm this by functional studies. Given the similar ROS concentration in NG- and HG-treated myotubes, this may explain why the functional studies did not indicate the PPP to be an explanatory factor.

For the first time, we observed that HG affected the oxidation of lactic acid in addition to affecting the oxidation of glucose and OA [6,7]. We have previously observed that treatment of myotubes with 10 mM lactate for four days induced the same suppression on glucose uptake as 20 mM glucose for four days (HG) [6]. In line with previous observations [6], we also observed a higher lactate concentration in the media from HG myotubes compared to cell medium from NG myotubes. This suggests that the oversupply of glucose pushes the glycolytic pathway. This is in line with the pathway analysis that indicated that HG affected glycolysis/gluconeogenesis in the cells. This was also confirmed by functional studies using Seahorse, where we observed that HG myotubes had higher glycolytic ATP production, basal glycolysis, basal PER, and PER derived from glycolysis compared to NG myotubes. These results may, at least in part, explain the effects of HG on energy metabolism.

## 5. Conclusions

Chronic hyperglycemia impaired substrate oxidation in cultured skeletal muscle cells. Although microarray gene expression analysis indicated several upregulated metabolic pathways, including glycolysis/gluconeogenesis, PPP, pyruvate metabolism, and *n*-glycan biosynthesis, functional studies only confirmed the role of glycolysis. Glycolytic ATP production, basal glycolysis, basal PER, and PER derived from glycolysis were all higher in HG than NG myotubes. The lactate concentration was also higher in media from cells exposed to chronic HG compared to NG control cells, and as lactic acid previously has been shown to reduce substrate oxidation, glycolysis and lactic acid may contribute, at least partly, to the hyperglycemia-induced decrease of substrate oxidation.
